# Retinal layer assessments as potential biomarkers for brain atrophy in the Rhineland Study

**DOI:** 10.1038/s41598-022-06821-4

**Published:** 2022-02-17

**Authors:** Matthias M. Mauschitz, Valerie Lohner, Alexandra Koch, Tony Stöcker, Martin Reuter, Frank G. Holz, Robert P. Finger, Monique M. B. Breteler

**Affiliations:** 1 Population Health Sciences, German Center for Neurodegenerative Diseases (DZNE), Venusberg-Campus 1/99, 53127 Bonn, Germany; 2 Department of Ophthalmology, University Hospital Bonn, Bonn, Germany; 3 MR Physics, German Center for Neurodegenerative Diseases (DZNE), Bonn, Germany; 4 Department of Physics and Astronomy, University of Bonn, Bonn, Germany; 5 Image Analysis, German Center for Neurodegenerative Diseases (DZNE), Bonn, Germany; 6 Institute for Medical Biometry, Informatics and Epidemiology, Faculty of Medicine, University of Bonn, Bonn, Germany

**Keywords:** Biomarkers, Health care, Signs and symptoms

## Abstract

Retinal assessments have been discussed as biomarkers for brain atrophy. However, available studies did not investigate all retinal layers due to older technology, reported inconsistent results, or were based on small sample sizes. We included 2872 eligible participants of the Rhineland Study with data on spectral domain–optical coherence tomography (SD–OCT) and brain magnetic resonance imaging (MRI). We used multiple linear regression to examine relationships between retinal measurements and volumetric brain measures as well as fractional anisotropy (FA) as measure of microstructural integrity of white matter (WM) for different brain regions. Mean (SD) age was 53.8 ± 13.2 years (range 30–94) and 57% were women. Volumes of the inner retina were associated with total brain and grey matter (GM) volume, and even stronger with WM volume and FA. In contrast, the outer retina was mainly associated with GM volume, while both, inner and outer retina, were associated with hippocampus volume. While we extend previously reported associations between the inner retina and brain measures, we found additional associations of the outer retina with parts of the brain. This indicates that easily accessible retinal SD-OCT assessments may serve as biomarkers for clinical monitoring of neurodegenerative diseases and merit further research.

## Introduction

The neurosensory retina and the brain derive from the same neural tissue, share morphologic and physiologic similarities and maintain direct synaptic connections over the whole life span^1^. To date, retinal layer thickness measurements with spectral domain–optical coherence tomography (SD–OCT) have become routine clinical biomarkers for ophthalmic diseases such as glaucoma^2,3^. Moreover, SD-OCT assessments are emerging as potential biomarkers for a variety of neurological and neurodegenerative diseases such as multiple sclerosis (MS) and dementia^4–8^. Previous studies reported associations between SD–OCT assessed retinal structures and magnetic resonance imaging (MRI) assessed brain parameters in small samples of mostly cognitively impaired participants^9,10^. Retinal imaging is non-invasive, easily assessable and less costly than MRI imaging, and therefore potentially suitable for mass screening. This, however, requires further elucidation of the relation of different retinal layers with brain features in the general population. To date, only one study evaluated the relation between retina layer parameters and the brain on a large-scale population level. This study used 1.5-Tesla (T) MRI which has since been superseded by the more advanced 3-T MRI. Furthermore, the authors concentrated on the inner retina and did not consider the outer retina due to technical limitations of retinal imaging at the time^11,12^.

Nowadays, more advanced segmentation algorithms and higher resolution imaging enable the automated and precise identification of additional retinal layers including the outer retina as well as novel and more precise peripapillary retinal nerve fiber layer (pRNFL) parameters such as Bruch’s membrane opening–minimum rim width (BMO–MRW) around the optic disk^13^. In addition, fractional anisotropy (FA) as assessed using diffusion tensor imaging (DTI), which is an indicator of white matter (WM) integrity, has recently been reported to correlate with disease severity in dementia and suggested as potential biomarker for neurodegeneration^14,15^. Thus, FA measures should be included in any future studies assessing the relationship between retinal and cerebral parameters.

Against this background we investigated the association of all retinal layers as well as novel peripapillary RNFL parameters with cerebral structural measures, including DTI derived FA, in a general, mostly Caucasian, population.

## Methods

### Study population

This study is based on the Rhineland Study, a community based prospective cohort study to which all inhabitants of two geographically defined areas in the city of Bonn, Germany, who are 30 years of age or older, are being invited. Persons living in those areas are predominantly German with Caucasian ethnicity. Participation in the study is possible by invitation only. The only exclusion criterion is insufficient German language skills to give informed consent. The study adheres to the tenets of the Declaration of Helsinki and has approval of the ethical committee of the Medical Faculty of the University of Bonn. All participants gave written informed consent. Our analyses are based on individuals who completed MRI and SD–OCT data and have complete data on covariables. Furthermore, as these diseases may have an impact on retinal SD-OCT assessments or cause non-degenerative brain changes, we excluded participants with a self-reported history of stroke, multiple sclerosis (MS), glaucoma, and macular degeneration or missing data on this (n = 50, n = 19, n = 62, and n = 75, respectively).

### Ophthalmic assessments and covariates

We assessed retinal layers of the right eye with our previously reported protocol using the Spectralis SD–OCT (Heidelberg Engineering, Heidelberg, Germany)^13^. In short, the in-built segmentation algorithms of the Heidelberg Eye Explorer (HEYEX) enables the automatic delineation of all macular layers including the outer retina: the inner retinal layers comprise the retinal nerve fiber layer (mRNFL), ganglion cell layer (mGCL), inner plexiform layer (mIPL), and inner nuclear layer (mINL). The outer retinal layers include the outer plexiform layer (mOPL), outer nuclear layer (mONL), and retinal pigment epithelium (mRPE). Around the optic disc, the algorithm delineates pRNFL and BMO–MRW which has shown good agreement with manual segmentation^16^. As initial quality assurance process, we filtered out large outliers and manually checked them. We excluded cases of pathology (e.g. epiretinal membrane) that deteriorate the reliability of the assessments. Refraction and best-corrected visual acuity (BCVA) were measured with an automated refractometer (Ark-1s, NIDEK CO., Tokyo, Japan). IOP was measured using non-contact tonometry (TX-20, Canon, Tokyo, Japan). Spherical equivalent (SE) was calculated as the spherical value and half of the cylindrical value. In absence of contraindications, participants were dilated for imaging using standard mydriatic agents (tropicamide and phenylephrine)^13^. Hypertension was defined as measured systolic blood pressure (SBP) > 139 mmHg and/or diastolic blood pressure (DBP) > 89 mmHg and/or use of antihypertensive drugs; diabetes was defined as measured fasting glycated hemoglobin (HbA1c) > 6.5% and/or the use of antidiabetic drugs. Body-mass-index (BMI) was defined as measured body weight (in kilogram) divided by square body height (in meters).

### Structural brain MRI

We performed MRI using a 3-T Siemens MAGNETOM Prisma MRI scanner (Siemens Healthcare, Erlangen, Germany) in absence of contraindications. Whole brain T1-weighted multi-echo magnetisation prepared rapid gradient-echo (MEMPRAGE, 0.8 mm isotropic resolution)^17,18^ images were acquired on two 3-T Siemens MAGNETOM Prisma MRI scanners (Siemens Healthcare, Erlangen, Germany) in absence of contraindications. Total brain volume (TBV), total hippocampal volume (THV) as well as total and occipital grey (GM) and white matter (WM) volumes, based on the Desikan-Killiany atlas, were automatically determined using FreeSurfer version 6.0, which has been shown to be extremely reliable with excellent test–retest intraclass correlation coefficients across different MRI scanners and sequences^19,20^.

### Diffusion MRI

Simultaneous-multi-slice diffusion weighted MRI (dMRI) was performed with a spin-echo echoplanar imaging (SE-EPI) sequence applying threefold slice-acceleration^21–23^. A compressed sensing^24^ diffusion spectrum imaging^25^ (CS-DSI) protocol^26^ was used to collect dMRI scans at 1.5 mm isotropic spatial resolution. After correction of susceptibility-induced^27^ and eddy-current-induced geometric distortions and subject motion^26,28^ using FSL version 6.0 (www.fmrib.ox.ac.uk/fsl) and after CS reconstruction^26,29^, fractional anisotropy (FA) was obtained from the diffusion tensor model^30^ using the MDT framework^31^. The Freesurfer processed T1-weighted MR image was used to generate a whole brain WM mask, which was further corrected for WM hyperintensities obtained from a T2- weighted FLAIR image, thresholded at an FA value of 0.3, constrained to avoid partial voluming with CSF^32^ and refined through FA skeletonization. Applying this mask, global FA values were computed as the average across voxels within normal appearing WM. Additionally, a WM tract-specific mean FA measure was derived for the optic radiation provided by the Jülich histological atlas^33^.

### Data analyses

Of the first 5000 participants, 3505 underwent MRI. Of these, 3395 participants had complete data on SD–OCT, and 3113 on all (co-)variables. The most frequent reason for missing SD–OCT data was technical issues, followed by low compliance during imaging resulting in low image quality. SD-OCT scans of 35 participants did not meet our predefined minimum quality standard of ≥ 20 dB signal strength (out of possible 40 dB) and hence were excluded from the analyses, leaving us with 2872 participants for final analyses. FA data for global WM and for the optic radiation were available in 2797 participants.

We used multivariable regression analyses to quantify the relations between SD-OCT measurements of specific retinal layers and MRI derived brain assessments. For ease of comparison, we standardized the different brain parameters. Hence, the regression models indicate percentage of difference in volume for each cerebral parameter. Moreover, we calculated beta-coefficients for each retinal layer per unit (mm^3^ and µm, respectively) as well as per standard deviation (SD). We adjusted for several previously reported confounders and determinants of retinal SD-OCT assessments^13,34^. Multivariable regression models included the brain assessments as outcome and the respective retinal layer as independent variable and were adjusted for age, sex, SE, estimated total intracranial volume (eTIV), BMI, hypertension, diabetes, and smoking. All analyses were performed with the statistical software RStudio (R version 4.0.3, RStudio, Inc, Boston, MA, https://www.rstudio.com/).

## Results

Compared to participants who underwent MRI (n = 3505), those without MRI data (n = 1495) were slightly older (57.7 ± 14.6 vs. 54.8 ± 13.6 years), included less women (52.9% vs. 58.0%), and showed a more positive SE (− 0.55 ± 2.6 vs. − 0.67 ± 2.6 diopters). The 2872 participants that were included in our final analysis showed similar age (53.8 ± 13.2 years), sex distribution (57.4% women), and SE (− 0.65 ± 2.5 diopters; Table 1), as the total group of participants with MRI.

**Table 1:** Characteristics of participants included in the analyses (n = 2872).

	Mean ± SD or n(%)
Age (years)	53.8 ± 13.2
Women	1649 (57.4%)
Spherical equivalent (dpt)	− 0.65 ± 2.5
Body-Mass-Index (kg/m^2^)	25.6 ± 4.2
**Smoking status**	
Never	1390 (48.4%)
Former	1103 (38.4%)
Current	379 (13.2%)
Hypertension	980 (34%)
Diabetes mellitus	116 (4%)
**Brain volumetric parameters (in ml)**	
Total brain	1111.0 ± 116.1
Total grey matter	461.2 ± 47.5
Total white matter	458.2 ± 57.8
Occipital grey matter	48.3 ± 6.2
Occipital white matter	42.4 ± 6.2
Hippocampi	7.92 ± 0.9
Fractional anisotropy (FA) of total white matter^a^	0.629 ± 0.02
FA of WM of the optic radiation^a^	0.628 ± 0.02
**Macular volumetric parameters (in mm**^**3**^**)**	
Retinal nerve fiber layer (mRNFL)	0.95 ± 0.12
Ganglion cell layer (mGCL)	1.08 ± 0.10
Inner plexiform layer (mIPL)	0.89 ± 0.07
Inner nuclear layer (mINL)	0.95 ± 0.06
Outer plexiform layer (mOPL)	0.82 ± 0.07
Outer nuclear layer (mONL)	1.73 ± 0.19
Retinal pigment epithelium (mRPE)	0.39 ± 0.04
Total retina	8.66 ± 0.39
**Optic disc thickness parameters (in microns)**	
Peripapillary retinal nerve fiber layer (pRNFL)	100.0 ± 10.8
Bruch’s membrane opening–minimum rim width (BMO–MRW)	341.3 ± 61.3

^a^n = 2797.

All inner and outer retinal layers except for mONL were positively associated with total brain volume (TBV). The associations were most outspoken for the inner retina, and stronger with WM than with GM volume (see beta-coefficients in Table 2). We saw a similar pattern of associations between retinal layers and occipital lobe volumes, with the estimates being even stronger than for total brain volumes (see beta-coefficients in Table 3). The inner retina around the optic nerve head (pRNFL and BMO–MRW) was similarly associated with brain volumetric measures as the macular inner retina. The mRPE, as part of the outer retina, was strongly associated with TBV and GM, but not with WM volume. The pattern of associations between retinal layer measures and the hippocampi, was similar to what we observed for total brain volumes, but relative effect sizes were much larger (Table 3). We found no association of mONL with any brain volume. Regarding the DTI analyses, mainly the inner retina showed positive associations with FA of normal appearing WM. This association was even stronger for FA of the optic radiation (see beta-coefficients in Table 4). As sensitivity analyses, we repeated all models stratified by age above and below 58 years (data not shown). We found similar, although smaller associations and some weaker associations vanished, particularly in the older group, in which we additionally found an association of ONL with total WM.

**Table 2:** Associations of retinal layers with brain volumetric parameters (n = 2872).

	Total brain volume			Total grey matter volume			Total white matter volume		
	Difference (%) per mm^3^/µm [95% CI]	Difference (%) per SD [95% CI]	p	Difference (%) per mm^3^/µm [95% CI]	Difference (%) per SD [95% CI]	p	Difference (%) per mm^3^/µm [95% CI]	Difference (%) per SD [95% CI]	p
mRNFL	1.58 [0.43; 2.74]	0.19 [0.05; 0.33]	0.007	0.34 [-1.04; 1.72]	0.04 [-0.13; 0.21]	0.63	2.89 [1.01; 4.76]	0.35 [0.12; 0.58]	0.002
mGCL	7.08 [5.61; 8.55]	0.70 [0.56; 0.85]	< 0.001	5.45 [3.67; 7.22]	0.54 [0.37; 0.72]	< 0.001	8.58 [6.17; 10.99]	0.86 [0.62; 1.10]	< 0.001
mIPL	8.61 [6.68; 10.55]	0.64 [0.49; 0.78]	< 0.001	6.94 [4.60; 9.27]	0.51 [0.34; 0.69]	< 0.001	9.89 [6.72; 13.07]	0.73 [0.50; 0.97]	< 0.001
mINL	3.17 [0.96; 5.39]	0.21 [0.06; 0.35]	0.005	0.85 [-1.80; 3.50]	0.05 [-0.12; 0.23]	0.53	4.46 [0.86; 8.06]	0.29 [0.05; 0.52]	0.02
mOPL	2.76 [0.87; 4.66]	0.20 [0.06; 0.33]	0.004	2.11 [-0.15; 4.38]	0.15 [-0.01; 0.31]	0.07	3.37 [0.28; 6.45]	0.24 [0.02; 0.46]	0.03
mONL	0.41 [-0.33; 1.15]	0.07 [-0.06; 0.22]	0.28	0.73 [-0.16; 1.62]	0.14 [-0.03; 0.31]	0.10	-0.24 [-1.45; 0.96]	-0.04 [-0.27; 0.18]	0.69
mRPE	5.89 [2.01; 9.77]	0.21 [0.07; 0.34]	0.003	6.59 [1.95; 11.22]	0.23 [0.07; 0.40]	0.005	3.99 [-2.33; 10.31]	0.14 [-0.08; 0.36]	0.22
pRNFL	0.04 [0.03; 0.06]	0.49 [0.35; 0.64]	< 0.001	0.02 [0.01; 0.04]	0.25 [0.08; 0.42]	0.004	0.06 [0.04; 0.08]	0.61 [0.38; 0.84]	< 0.001
BMO–MRW	0.00 [0.00; 0.01]	0.25 [0.10; 0.39]	< 0.001	0.00 [0.00; 0.00]	0.00 [-0.18; 0.16]	0.92	0.01 [0.00; 0.01]	0.42 [0.19; 0.65]	< 0.001

*mRNFL* macular retinal nerve fiber layer, *mGCL* macular ganglion cell layer, *mIPL* macular inner plexiform layer, *mINL* macular inner nuclear layer, *mOPL* macular outer plexiform layer, *mONL* macular outer nuclear layer, *mRPE* macular retinal pigment epithelium, *pRNFL* peripapillary RNFL, *BMO–MRW* Bruch’s membrane opening–minimum rim width.

Macular layer volumes per mm^3^; pRNFL and BMO–MRW per µm.

**Table 3:** Associations of retinal layers with occipital and hippocampal volumetric parameters (n = 2872).

	Occipital grey matter volume			Occipital white matter volume			Total hippocampal volume		
	Difference (%) [95% CI] per mm^3^/µm	Difference (%) per SD [95% CI]	p	Difference (%) [95% CI] per mm^3^/µm	Difference (%) per SD [95% CI]	p	Difference (%) per mm^3^/µm [95% CI]	Difference (%) per SD [95% CI]	p
mRNFL	2.41 [-0.50; 5.31]	0.29 [-0.06; 0.64]	0.10	5.93 [2.53; 9.35]	0.72 [0.31; 1.14]	< 0.001	2.64 [0.14; 5.13]	0.32 [0.02; 0.62]	0.04
mGCL	12.47 [8.74; 16.20]	1.24 [0.87; 1.62]	< 0.001	15.00 [10.61; 19.39]	1.50 [1.06; 1.93]	< 0.001	13.27 [10.08; 16.47]	1.32 [1.01; 1.64]	< 0.001
mIPL	13.77 [8.86; 18.68]	1.02 [0.66; 1.38]	< 0.001	15.18 [9.40; 20.97]	1.13 [0.70; 1.55]	< 0.001	17.46 [13.26; 21.65]	1.29 [0.98; 1.60]	< 0.001
mINL	-1.95 [-7.53; 3.63]	-0.13 [-0.49; 0.24]	0.49	2.35 [-5.50; 8.91]	0.15 [-0.27; 0.58]	0.43	6.94 [2.16; 11.73]	0.45 [0.14; 0.76]	0.004
mOPL	-0.32 [-5.10; 4.45]	-0.02 [-0.37; 0.32]	0.89	0.11 [-7.34; 5.73]	0.00 [-0.01; 0.01]	0.97	5.78 [1.68; 9.88]	0.42 [0.12; 0.71]	0.005
mONL	1.74 [-0.12; 3.61]	0.33 [-0.02; 0.69]	0.07	-0.10 [-2.30; 2.09]	-0.02 [-0.04; 0.04]	0.93	-0.25 [-1.86; 1.35]	-0.05 [-0.35; 0.26]	0.76
mRPE	4.51 [-5.29; 14.29]	0.16 [-0.19; 0.50]	0.37	3.68 [-7.83; 15.18]	0.13 [-0.28; 0.54]	0.53	11.14 [2.75; 19.54]	0.39 [0.10; 0.69]	0.009
pRNFL	0.14 [0.11; 0.17]	1.49 [1.13; 1.84]	< 0.001	0.19 [0.15; 0.22]	2.00 [1.59; 2.41]	< 0.001	0.10 [0.07; 0.13]	1.06 [0.76; 1.39]	< 0.001
BMO–MRW	0.01 [0.00; 0.01]	0.56 [0.21; 0.92]	0.02	0.02 [0.01; 0.02]	0.96 [0.54; 1.38]	< 0.001	0.01 [0.00; 0.01]	0.48 [0.17; 0.78]	0.002

*mRNFL* macular retinal nerve fiber layer, *mGCL* macular ganglion cell layer, *mIPL* macular inner plexiform layer, *mINL* macular inner nuclear layer, *mOPL* macular outer plexiform layer, *mONL* macular outer nuclear layer, *mRPE* macular retinal pigment epithelium, *pRNFL* peripapillary RNFL, *BMO–MRW* Bruch’s membrane opening–minimum rim width.

Macular layer volumes per mm^3^; pRNFL and BMO–MRW per µm.

**Table 4:** Associations of retinal layers with white matter fractional anisotropy (n = 2797).

	FA total white matter			FA optic radiation		
	Difference (%) per mm^3^/µm [95% CI]	Difference (%) per SD [95% CI]	p	Difference (%) per mm^3^/µm [95% CI]	Difference (%) per SD [95% CI]	p
mRNFL	0.25 [-0.11; 1.40]	0.08 [-0.01; 0.17]	0.09	2.72 [1.62; 3.82]	0.33 [0.20; 0.46]	< 0.001
mGCL	2.31 [1.34; 3.28]	0.23 [0.13; 0.27]	< 0.001	6.38 [4.98; 7.78]	0.64 [0.50; 0.78]	< 0.001
mIPL	2.92 [1.64; 4.19]	0.22 [0.12; 0.31]	< 0.001	7.22 [5.37; 9.07]	0.53 [0.40; 0.67]	< 0.001
mINL	1.88 [0.43; 3.32]	0.12 [0.03; 0.22]	0.01	3.01 [0.90; 5.13]	0.20 [0.06; 0.33]	0.005
mOPL	1.29 [0.05; 2.52]	0.09 [0.01; 0.18]	0.04	1.85 [0.04; 3.66]	0.13 [0.00; 0.26]	0.05
mONL	0.34 [-0.15; 0.82]	0.06 [-0.03; 0.16]	0.17	0.85 [0.14; 1.56]	0.16 [0.03; 0.30]	0.02
mRPE	-0.30 [-2.84; 2.24]	-0.01 [-0.10; 0.08]	0.82	1.01 [-2.71; 4.72]	0.04 [-0.10; 0.17]	0.60
pRNFL	0.01 [0.00; 0.02]	0.14 [0.04; 0.23]	0.004	0.06 [0.04; 0.07]	0.59 [0.46; 0.73]	< 0.001
BMO–MRW	0.00 [-0.01; 0.01]	-0.08 [-0.17; 0.01]	0.10	0.00 [0.00; 0.00]	0.06 [-0.07; 0.20]	0.36

*FA* fractional anisotropy, *mRNFL* macular retinal nerve fiber layer, *mGCL* macular ganglion cell layer, *mIPL* macular inner plexiform layer, *mINL* macular inner nuclear layer, *mOPL* macular outer plexiform layer, *mONL* macular outer nuclear layer, *mRPE* macular retinal pigment epithelium, *pRNFL* peripapillary RNFL, *BMO–MRW* Bruch’s membrane opening-minimum rim width.

Macular layer volumes per mm^3^; pRNFL and BMO–MRW per µm.

## Discussion

We found associations of volume/thickness of inner retinal layers with TBV, which seemed to be primarily driven by a relationship with WM. These associations were even stronger within the occipital lobe and with the hippocampus, and were also found in the inner retina around the optic disc (pRNFL and BMO–MRW). That the relation was primarily with WM was supported by our finding of significant relationships between those layers with DTI parameters of WM integrity. In contrast, we observed a relation of parts of the outer retina with TBV, which seemed to be due to an association with GM. Our data suggest SD-OCT-derived retinal assessments can potentially serve as biomarkers of cerebral atrophy.

Most of the few published studies on the relation of MRI brain assessments and OCT-derived retinal layer measurements were based on small cohorts of selected participants and included both, neurologically healthy and diseased individuals. These studies reported diverse and partially conflicting results on the relation of the inner retina, mostly pRNFL and mGCL, and different cerebral areas^9,10,35,36^. The limited power in, and the huge methodological differences between, most of these studies, however, hinders drawing strong conclusions on the basis of their results.

The only population-based study that we are aware of that reported on the relation between retinal measures and brain features, is the Rotterdam Study^11,12^. This study found strong associations of the innermost retinal layers (mainly pRNFL and mGCL and to some extent mIPL) with both, GM and WM volumes and partly with FA as assessed using DTI^11,12^. However, in the Rotterdam Study, segmentation algorithms were only able to distinguish the inner retina. In their analysis, the mIPL was the most outer, and thus potentially least precisely, segmented layer. Our data corroborate their findings, and extend those in that we found strong associations of mIPL with all cerebral assessments and additionally of mINL as further inner layer with TBV. Hence, we found that various layers of the inner retina both at the macula and the optic disc were associated with brain volumes, especially with WM volume.

We were able to also segment the outer retina, and found that the mRPE was associated with TBV, which seemed specifically driven by GM. We are only aware of two small studies that investigated the relation of the outer retina with MRI brain parameters. One study found no relation in 52 participants^37^, whereas the other study found that the outer retina was associated with TBV in 64 participants^38^. However, besides the small sample sizes, these studies did not delineate all retinal layers and only adjusted for age and sex as potential confounders within their analyses. Our findings therefore need confirmation from larger, methodologically robust studies.

A question is what underlies our findings. That we found the strongest associations for the inner retina may indicate that ganglion cells and their inter-connections (both, distal axons and proximal dendrites) decline in case of cerebral deterioration/changes. Previous studies hypothesized retrograde degeneration as a potential pathomechanism, leading from cortical atrophy via reduced neuronal inter-connections to decline of retinal axons^4,12,34^. However, this pathomechanism appears less likely for the outer retina. Here, systemic changes e.g. associated with impaired perfusion or vasculopathy may play a role. The mRPE is a thin layer of highly metabolically active cells supporting photoreceptors in the maintenance of the visual cycle, contributes to the blood-retina barrier^39^ and is not part of the neuro-sensory retina with direct connection to cerebral neurons. This may explain why we found no association of mRPE with WM volumes as we did for the inner retina. While associations of the neuro-sensory (inner) retina with the brain can be explained by the close anatomical connection, we speculate that the observed associations of mRPE are less direct and represent a more general metabolic dysregulation. Interestingly, we found no relation of the mONL with any cerebral assessment. The mONL represents a large layer between the inner retina and the mRPE and consists of parts of the photoreceptors. The lack of any association therefore suggests that photoreceptors, as the first cells within the visual afferent pathway, are relatively unaffected by cerebral changes.

We found the strongest associations, both for inner and outer retinal layers, with volumes of the hippocampi. Only few, small studies reported correlations of retinal measurements with hippocampus volumes and suggested these to be potential biomarkers of neurodegeneration^40–42^. As hippocampus volumes decrease early in cognitive impairment^43^, retinal assessments may be an easily accessible biomarker of hippocampus atrophy and cognitive decline^44^. Our data suggest that in clinical routine, SD-OCT assessments of retinal layers may be useful as additional examination in the longitudinal monitoring of neurological/neurodegenerative diseases, which e.g. has already been proposed for MS^45^.

Apart from structural measurements, we found various layers of the inner retina to be associated with FA, a proxy measure for WM microstructural integrity. As expected, effects were larger in the optic radiation as compared to total WM. This adds to and extends the results of the Rotterdam Study, which reported only the innermost layers (pRNFL and mGCL) to be associated with FA^12^.

To our knowledge, our study is the first to report on the relation of fully segmented retinal layers and various cerebral parameters from structural MRI and DTI in a large, general Caucasian population. The strengths of our study include the large community-based population with a wide age range from 30 to 94 years. The weaker associations in the sensitivity analysis are likely due to the reduced sample size and, in the older group, the general decrease in retinal layer thickness with age, which leads to smaller absolute differences and likely reduced accuracy in detecting changes. Moreover, we hypothesize the association of ONL with total WM to be spurious, as the ONL contains cell bodies, which are not likely directly connected to the axons of WM and we found no association of ONL with any MRI assessment in the total cohort. The study protocol comprised comprehensive and standardized ocular, MRI-based cerebral and systemic deep phenotyping. SD-OCT and MRI imaging were performed using a state-of-the-art device with high resolution scans and automated layer segmentation, including the deeper outer retinal layers^13^. For MRI image analysis we performed post-processing based on well-established atlases. Moreover, we adjusted our analyses for several ocular confounders, which has not been the case in many neurological studies on SD-OCT. Yet, several limitations must be considered. Even though participation was possible by invitation only, a self-selection of younger and more healthy participants may have occurred. Hence, our study population is not strictly representative of the entire German population. To the extent that this may have introduced bias, we consider it most likely that it led to an underestimation of effects. Furthermore, information on some diseases was self-reported which may have been less reliable than medical diagnoses. We did not manually check the retinal layer segmentation of all scans, but we excluded scans below a strict quality threshold. It is therefore unlikely, also given the large sample size, that remaining segmentation artefacts have confounded our results to any relevant extent^13^. Lastly, we performed a number of statistical models, which may have increased alpha error accumulation. However, we consider beta-coefficients and respective confidence intervals much more important than p-values as a binary cut-off in this exploratory study. The widely used Bonferroni correction (in our study 0.05/72 ≈ 0.00069) has frequently been reported to be over-conservative causing too many false negatives^46^. Hence, we report beta-coefficients, confidence intervals, and p-values so that the reader is able to evaluate both statistical significance and clinical relevance. In conclusion, we found strong associations of the inner and outer retina with various cerebral structures. Our study implies that SD-OCT assessments of different retinal layers may serve as potential biomarkers for cerebral atrophy and may aid in neurodegenerative disease monitoring. Further research to evaluate the value of SD-OCT assessments of the retina in detecting or monitoring cerebral changes and neurodegenerative diseases is warranted.

## Data Availability

The datasets analyzed during the current study are not publicly available due to participant privacy, but may be available from the corresponding author on reasonable request.
